# Implementation of a population-based epidemiological rare disease registry: study protocol of the amyotrophic lateral sclerosis (ALS) - registry Swabia

**DOI:** 10.1186/1471-2377-13-22

**Published:** 2013-02-17

**Authors:** Gabriele Nagel, Hatice Ünal, Angela Rosenbohm, Albert C Ludolph, Dietrich Rothenbacher

**Affiliations:** 1 Institute of Epidemiology and Medical Biometry, Ulm University, Helmholtzstr. 22, Ulm 89081, Germany; 2 Department of Neurology, Ulm University, Ulm, Germany

**Keywords:** Amyotrophic lateral sclerosis, Registry, Risk factors

## Abstract

**Background:**

The social and medical impact of rare diseases is increasingly recognized. Amyotrophic lateral sclerosis (ALS) is the most prevalent of the motor neuron diseases. It is characterized by rapidly progressive damage to the motor neurons with a survival of 2–5 years for the majority of patients. The objective of this work is to describe the study protocol and the implementation steps of the amyotrophic lateral sclerosis (ALS) registry Swabia, located in the South of Germany.

**Methods/Design:**

The ALS registry Swabia started in October 2010 with both, the retrospective (01.10.2008-30.09.2010) and prospective (from 01.10.2010) collection of ALS cases, in a target population of 8.6 million persons in Southern Germany. In addition, a population based case–control study was implemented based on the registry that also included the collection of various biological materials.

Retrospectively, 420 patients (222 men and 198 women) were identified. Prospectively data of ALS patients were collected, of which about 70% agreed to participate in the population-based case–control study. All participants in the case–control study provided also a blood sample. The prospective part of the study is ongoing.

**Discussion:**

The ALS registry Swabia has been implemented successfully. In rare diseases such as ALS, the collaboration of registries, the comparison with external samples and biorepositories will facilitate to identify risk factors and to further explore the potential underlying pathophysiological mechanisms.

## Background

A registry is an organized system that collects clinical and other data to a given purpose with methods of observational studies in a standardized manner [1]. Basically, it composites data concerning all cases of a particular disease or all subjects sharing specified characteristics. Ideally it is implemented in a defined population and may also include longitudinal information. The systematic collection of data allows evaluating specific outcomes, health-related characteristics and end points according to epidemiological criteria [1].

Different registries with a focus on disease, treatment, special patient or vulnerable groups (such as pregnant women) or outcome exist: procedure-related registries (centered on specific drugs, therapeutic, or diagnostic procedures), patient registries, or specific disease registries. Depending on the focus of the registry, the target population, data collection and methods will be chosen accordingly [2]. The selection of the target population depends also on the research question, which can be related to the epidemiology of the disease, course of disease and mortality, (adverse) effects of treatment or quality of life just to name a few. To date an impressive variety of medical registries exist [3,4].

During the past years, it is increasingly recognized that rare diseases are an important medical and public health issue [5]. Amyotrophic lateral sclerosis (ALS), the most frequent of the motor neuron diseases (MND), is such a rare disease with an estimated incidence of 2–3 cases per 100,000 person-years [6,7]. The majority of patients die within 3 to 5 years, mostly as a result of respiratory failure. The disease leads to a rapidly progressive damage of the motor neurons with subsequent muscle paralysis. ALS starts in 40-50% of patients in the upper extremities, usually initially with progressive atrophy and paralysis of the small hand muscles [6,7]. About 20-30% of patients show symptoms of the lower limbs at first signs of manifestation, increasing the risk for stumbling and as a consequence the risk for consecutive falls [6,7].

The diagnosis ALS relies on clinical criteria such as progressive paralysis, amyotrophy, hyperreflexia, and spasticity [8,9]. During the course of disease, dysphagia, dyspnea, depression, pain and sleep disorders can occur [6]. The treatment of ALS is based on symptomatic and pathogenetic orientated therapy. Although the combination of both approaches has led to a significantly better survival of ALS patients [6,9], the prognosis is yet poor.

There is evidence that smoking and physical activity is associated with ALS risk [6,10]. However, the etiology and pathogenesis of ALS is still largely unknown and its genetic component is only partially understood. Major gaps remain in the understanding of its pathogenesis with the basic principle of selective vulnerability and potentially resulting therapeutic consequences.

With this paper, we would like to describe the study protocol and the implementation steps of the amyotrophic lateral sclerosis (ALS) registry Swabia and the adjunct population-based case–control study. We also illustrate the potential of disease registries as efficient epidemiological approach for interdisciplinary and analytical research of rare diseases by combining the clinical registry data with information from an epidemiological population-based case–control study.

## Methods

### Mission of the ALS registry Swabia

The objectives of the population-based ALS registry Swabia is to determine the incidence, prevalence and mortality of ALS in a defined geographical region in the South of Germany. Furthermore, by means of a population-based case–control study, potential risk factors such as life time history of physical activity, sports, head injuries, and metabolic factors will be further investigated to name only a few risk factors which are in the focus. In addition, the collection of blood samples and other biological materials shall be used to analyze genetic and biological factors to elucidate the underlying pathophysiological mechanisms and to identify potential starting points for the development of new treatment approaches.

#### Study design and study population

**Figure 1:**
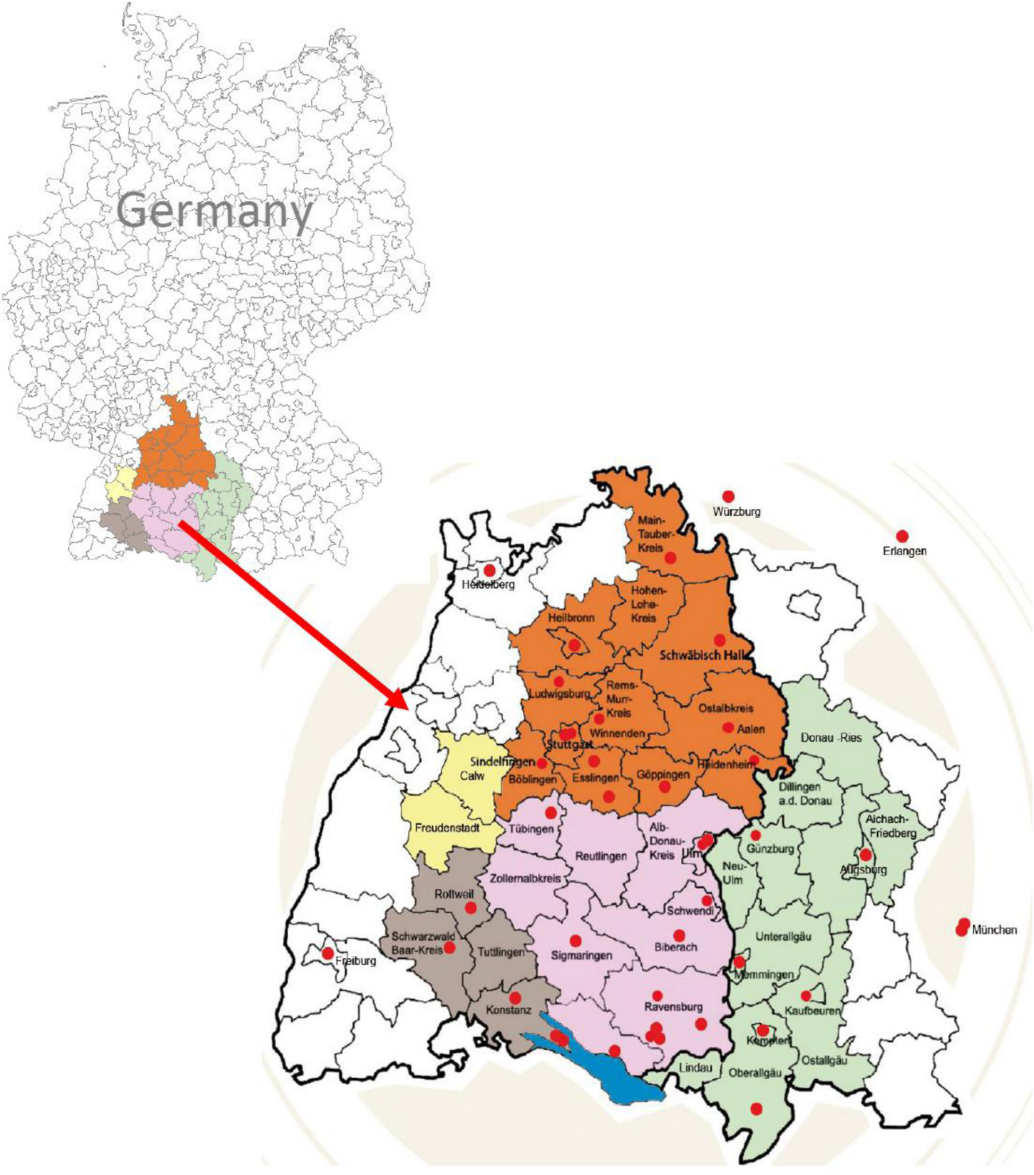
Study region of the ALS-registry Swabia located in the South of Germany.

The ALS registry Swabia is conducted as a clinical-epidemiological registry with the aim to collect data on all newly diagnosed ALS cases in the target population to estimate epidemiological data on incidence, patient and disease characteristics, and natural history of ALS. In addition, a case–control study is attached to investigate risk factors of ALS and factors of disease progression in an analytical setting. The study population consists of all inhabitants living in the area of Swabia as from the year 2008. The study region is defined by cities and counties that reflect the geographical or administrative borders of Swabia, located in the South of Germany (Figure 1).

#### Registry cases

The inclusion and exclusion criteria for the ALS registry are clearly defined by the diagnosis of ALS, the date of diagnosis and the residence at the time of diagnosis. ALS cases are defined by the El Escorial criteria. Depending on the onset of symptoms, following locational criteria can be distinguished: bulbar, cervical, thoracic, and lumbosacral and signs of upper or lower motor neurons. To confirm the diagnosis of ALS, the information is verified by neurologists using the internationally standardized "El Escorial criteria" [11,12]. Patients with suspected ALS are tracked and evaluated during the clinical course.

The regional cooperation partners identify patients and obtain informed consent, before they notify the ALS registry at the Ulm University. In the ALS registry, both case report forms (CRFs, Table 1) and electronic notes per Email or Fax are used. Information on the onset of ALS, the localization, diagnostic and treatment procedures are collected by study nurses from the medical records.

**Table 1:** Information collected and instruments used in ALS registry Swabia

**ALS**-**case report form**	**Items**	**Source**
Symptoms	Upper, lower extremities (distal, prox.), bulbar, spastic, cerebellar and further symptoms	According to EURALS
Localization	HSP, PLS, PMA, SMA, Bulbar, Bulbarparalysis, Flail- Arm-Syndrome, Flail- Leg-Syndrome	According to EURALS
Diagnosis	Date, proof	According to EURALS
Treatment	Past, current	According to EURALS
**Case**-**Control questionnaire**		
Sociodemographic status	Age, gender, school education, occupation	According to EURALS
Care / care givers		
Family history	Motor neuron diseases in first and second degree relatives	
Comorbidities	List of common chronic diseases and other diseases (history)	
Medication	Life-time history of anti-inflammatory drugs	
Smoking	Life-time history	According to EURALS
Alcohol consumption	Life-time history	According to EURALS
Diet	Diet and nutritional supplements	
Physical activity	Life-time history of sports and physical activities	According to EURALS
Trauma	Life-time history	According to EURALS
Injuries	Life-time history	According to EURALS
Reproductive history (for women)	Menstruation, hormons, pregnancy, menopause, operations	According to EURALS
Quality of life	SF 12	M. Morfeld, I. Kirchberger, M. Bullinger [13]
Subjective quality of life	SQoL	J. Bernheim (1999) [14]
Neuropsychiatric tests (Hospital Anxiety and Depression Scale)	HADS	C. Herrmann-Lingen, U. Buss, R. P. Snaith (1995) [15]
Bedside test to assess the function of the frontal brain (Frontal Assessment Battery)	FAB	Dubois et al. [16]
Montreal Cognitive Assessment	MoCA	Nasreddine et al. [17]
Coping Achievement Motivation Scale	AMS	J. W. B. Lang, S. Fries (2006) [18]

ALS is a clinically based diagnosis. In order to achieve standardization, all cases are reviewed by an experienced neurologist according to the El Escorial criteria [11,19]. Regular biannual meetings and special diagnostic courses are organized with all cooperation partners to strengthen the regional network and to ensure a high compliance with the standardized data collection procedures. An ALS registry homepage has been set up to inform about the purpose of the ALS registry Swabia, the data collection procedures and results of the registry [20].

#### Study design

**Figure 2:**
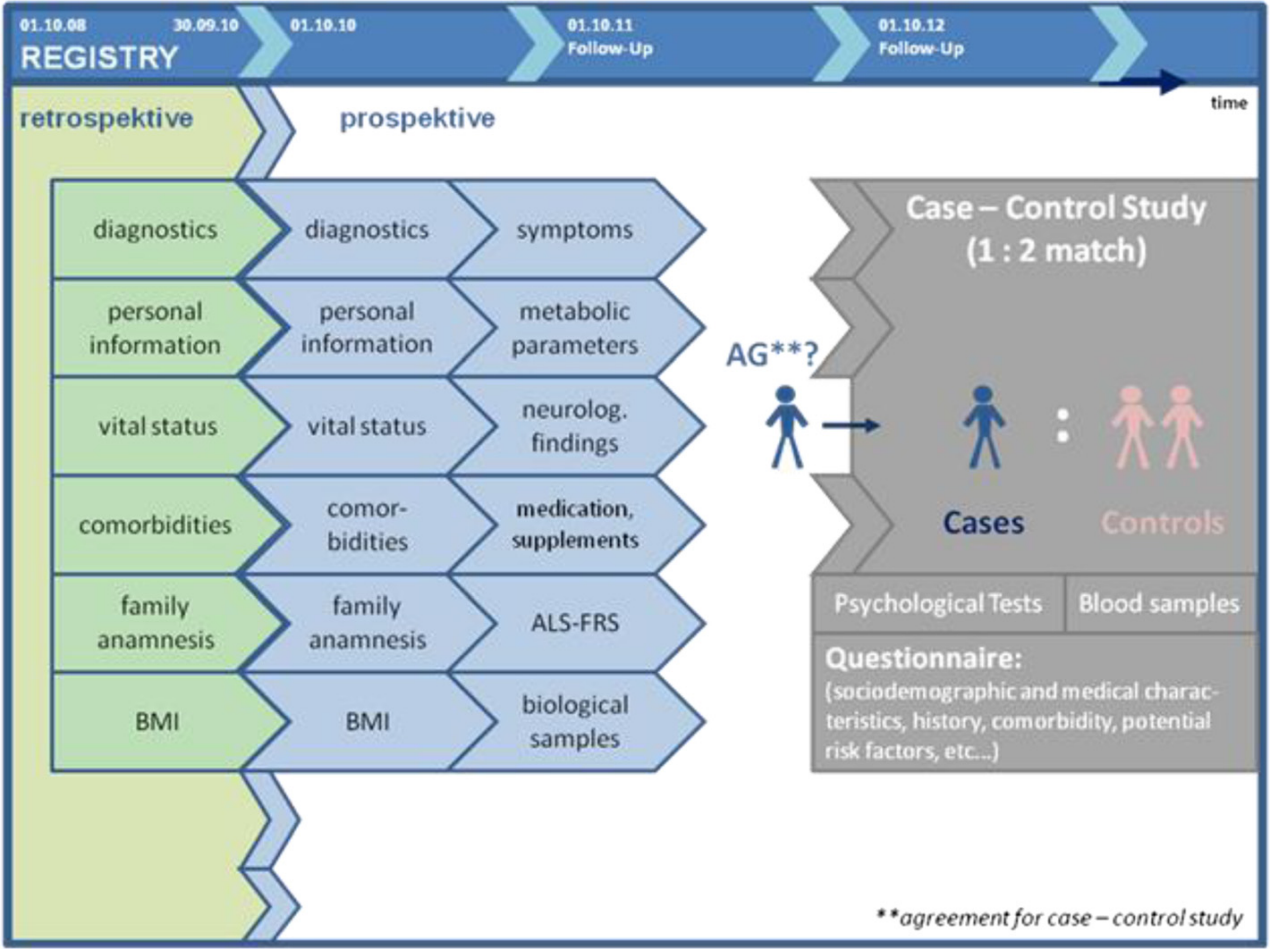
Recruitment procedures and collected information in the ALS registry Swabia and the attached case-control study.

The ALS registry comprises data for the descriptive analyses of onset and the natural history of ALS progression. The ALS registry functions as an observational study conducted as a population-based cohort study with a retrospective and a prospective part. The implementation of the ALS registry in Swabia started 1st of October 2010 with prospective collection of all newly diagnosed ALS cases in the target population. In addition, information on ALS cases diagnosed between 01.10.2008 until 30.09.2010 has been collected retrospectively (Figure 2). The retrospective branch has the aim to implement the registry infrastructure and management in the target area and to provide first estimates of the incidence in the study region. Regional collaboration partners contact ALS patients and ask for consent to send the information to the ALS registry at the Neurological University Hospital Ulm. To date 36 regional partners collaborate in the registry (see list at the end of the publication).

Patients from the prospective registry part diagnosed after 01.10.2010 were also asked to provide informed consent to participate in a population-based case–control study in order to investigate risk factors of ALS. Control subjects are selected in the region by a random sample of the corresponding registration office, contacted and invited to participate in the case–control study. To each case, two sex and age frequency-matched controls are randomly sampled from the general population on the relevant catchment area of the case. The potential controls are invited to participate by mail and in case of participation informed consent is obtained.

### Data collection and quality assurance

#### ALS registry

The data collection office of the ALS registry Swabia is located in the Department of Neurology at the Ulm University to which new cases are reported, checked eligibility for inclusion and were the encryption of personal data is performed. The data coordinating and analysis center is located at the Institute of Epidemiology and Medical Biometry where only pseudomyzed data of the cases are stored. The identification and recruitment of the age, gender and region matched controls is also organized from here. Internal quality checks and analyses are also performed on a routine basis in the data coordinating and analysis center.

#### Case–control study

Patients and controls who provided their informed consent to participate in the case–control study will be visited by a study nurse for an interview, neuropsychological tests and blood sampling. The instruments and tests are identically conducted in both groups (Table 1) and have been adapted to the instruments used by the European ALS group EURALS [21].

#### Biological samples

Blood sample (EDTA-plasma, serum, PAXgene) are collected from cases and controls in a standardized manner. EDTA-plasma and serum are obtained by centrifugation for 10 min at 2000 × g and 4°C. PAXgene samples were processed within the same day according to the standard protocol. Blood specimens were transferred into 0.5-1.0 ml sample containers with screw tops and were stored for analysis of biomarkers of exposure and susceptibility. ALS patients are asked to allocate dispensable material from routine examinations such as cerebral spinal fluid and muscle biopsies for the biobank which is associated with the ALS registry. Blood samples and specimens were stored for future analysis of biomarkers of exposure and genetic analyses in -80° Celsius freezers or in liquid nitrogen.

#### Follow-up

For all cases of the case–control study active follow-up is conducted one year after inclusion into the study. Data on the course of disease and quality of life is collected by a standardized questionnaire. A study nurse visits the patients to perform neuropsychological tests and to collect a blood sample. Instruments used in the follow-up are based on the baseline examination and include questions on respiratory aids and parenteral nutrition (Table 1). Information of the vital status of participants will be collected from all participants. If a patient deceased the respective death certificate will be collected from the regional public health office. In Germany, the location of death and not the location of the last place of residence is determining in which local health office the death certificate is stored. The main cause of death will be coded according to the coding rules of the German mortality statistics. In addition, information on comorbid conditions will be collected.

#### Statistical methods and analysis plans

The size of the target population has been estimated by power calculation for the registry-based case–control study. Under the assumption of an ALS incidence of 2 to 3 cases per 100,000 person-years, we estimated that a target population of about 8.6 million inhabitants will be sufficient to collect data from 200–240 patients per year [21].

For the associated case–control study, we estimated that about 75% of patients will participate. Under the assumptions of a significance level of two-sided α = 0.05, power calculation revealed that the power of the study will be large enough for major exposure variables to detect an association of an odds ratio of two or larger with a power of 80% (assuming a sample size of 315 ALS cases and 630 control cases).

Descriptive epidemiological measures, such as prevalence and incidence of ALS, will be estimated from the ALS registry data (Table 2). These measures will also be estimated for small geographic areas to investigate potential differences. Comparison with data collected in the EURALS project [22] will be performed in combination with internal, external or historical comparison groups. Standardized rates and relative risks can be calculated. Besides descriptive epidemiological measures, explorative analyses and the testing of hypothesis can be performed. Table 2 shows the objectives and epidemiological measures obtained from the clinical ALS registry and additional information derived from the attached case–control study.

**Table 2:** **Objectives and epidemiological measures obtained from the clinical ALS**-**registry and additional information derived from the attached case**–**control study**

**a. Clinical ALS registry (patient centered analyses)**	
Descriptive measures:	-ALS prevalence (proportion)
	-Symptoms at disease manifestation
	-Distribution of localization at onset
	-Time from first symptoms to clinical diagnosis (diagnostic delay)
	-Distribution of diagnostic procedures in routine clinical care of ALS
	-Application of therapeutic means and measures during follow-up
	-Background rate of adverse events in ALS population
	-Rate and risk factors of comorbid diseases in ALS population
	-Natural history of disease (progression free survival, overall survival, case fatality rate)
	-Quality of life
Analytical measures:	-(Long-term) efficacy of therapies under routine clinical care conditions on various outcomes (progression free survival, overall survival) and occurrence of adverse events (safety outcomes)
	-Identification of prognostic markers of disease progression (includes assessment of potential new drug targets
**b. Epidemiological ALS Registry (includes geographical analyses)**	
Descriptive measures:	-All of the above mentioned (see 2.a), additionally
	plus incidence
	plus mortality
	plus geographic and frequency distribution of risk factors
Analytical measures:	-see 2.a
	plus spatial changes
	plus cluster analysis
	-in nested case – control study
	plus investigation of risk factors and potential causes of disease
Molecular epidemiology:	-in biobanked material
	plus investigation of pathogenetic pathways (blood samples)

In the year 2013, according to the power analysis the number in the case–control study will be sufficiently large to start the analyses on potential risk factors. Previously we will evaluate the completeness of the ALS-registry Swabia by calculation of the ALS incidence in Swabia, comparison with other data sources and applying spatial scan statistics. For known exposure factors in the target population, relative and attributable risks can be calculated. Additional clinical data during follow-up will facilitate to describe the course of disease and the outcome of the disease during the clinical course of different phenotypes and to determine the survival time.

#### Ethical considerations

Data protection is a prerequisite of a registry, which includes transparency of the data collection and analyses. Informed consent is necessary for the collection of personal data. Withdrawal of the data should be possible at any time. International, national and state rules have been considered implementing the ALS registry Swabia. Approvals of the ethical committees of the Ulm University, medical association (Landesärztekammer) of the state of “Baden-Württemberg”, and the medical association of the state of “Bayern” have been obtained.

In the target region, 36 neurological departments and clinics are cooperating to notify newly diagnosed ALS cases to the ALS registry Swabia. In addition, five adjacent neurological centers, not directly located in the study region, but potentially receiving cases for diagnosis, care and treatment of the study region are visited annually in order to identify all patients from the target population. Therefore a complete coverage of all medical centers specialized in the diagnosis and treatments of ALS are included.

#### Implementation

Regional cooperation partners report new cases to the data collection office at the Neurological Clinic, where tests for duplicates and the encryption of personal data are performed. So far, with the exception of two administrative regions, reporting of cases works very well. The infrastructure and the processes for the registry and case–control study have been implemented. Initially we informed the public, the scientific community and physicians in the region about the ALS registry in Swabia. Before starting with the implementation of the ALS-registry, a pilot study among 10 patients was performed in order to test the instruments and the organization of the ALS registry Swabia. Based on the helpful experiences made in the pilot study, the organization and the instruments were modified accordingly. Standard operation procedures were written and distributed among the study staff. The study team has been trained concerning the general study procedures and specific tests such as neuropsychiatric tests in order to work according to principles of good scientific and epidemiological practice.

The definitions of core data concerning ALS diagnosis and phenotype, which have to be collected in all clinics and of non-core data, have been defined in order to guarantee the statistical analyses. Variables for the core data are: sex, age, diagnosis, date of first symptoms and diagnosis, date of death and cause of death. Besides these variables, the type and the unit should be defined for the collection of the data (e.g. for length: numerical in m or cm). These recommendations are documented in a standard operation handbook. In the ALS registry Swabia, regular checks of the core data set and the end-point data have been implemented to sustain high quality data over the entire observation period.

#### Baseline study characteristics

##### ALS registry

Between October 2008 and end of September 2010, 420 incident ALS cases were identified (as is July 31, 2012). There were 53% men and 47% women among ALS cases.

##### Prospective data

Since 1st of October 2010, 188 cases were included into the registry for the case–control part of the study, 61% were men and 39% women. The first follow-up includes a self-administered standardized questionnaire, a personal interview including several neuro-psychological logical tests, and the collection of a blood sample. The prospective part of the ALS registry including case–control study and the collection of biomaterial is ongoing.

##### ALS case – control study

In the case–control study, 124 ALS patients agreed to participate and provided informed consent. According to the place of residence, age and gender of the patients, a random sample in the registration office is requested. The potential control persons are contacted and invited to participate in the study. After agreement, the study material is sent and a date for the visit of the study nurse is fixed. On average 10 randomly selected control subjects were selected, then contacted and the first 2 participating control subjects included. So far, data from 175 control persons have been collected. Notably, it is much more difficult to recruit healthy controls for the study and certain incentives should be included into the recruitment process to give patients and additional motivation. Most are not aware of their importance to help to combat this terrible disease. So far, comparison of the age and sex distributions in the case–control study with the retrospective and prospective ALS registry data revealed no indication of selection bias.

## Discussion

In this paper, we describe the rational, the study design and the basic characteristics of the ALS registry Swabia and the adjunct population-based ALS case–control study. We further discuss the prerequisites and the potentials of registries in the investigation of rare diseases. The epidemiological ALS registry Swabia has been successfully implemented in the target region covering about 8.6 million inhabitants. Retrospectively 420 incident ALS cases were identified, of whom 53% were men and 47% women. Since October 2010 data of 188 ALS cases have been collected prospectively.

Epidemiological registries record all newly diagnosed cases of illnesses in a geographically defined area. The main criterion for the inclusion in an epidemiological registry is the residence at diagnosis. For the ALS registry a core data set has been defined. As part of the data collection process has to be included in the routine clinical work of the collaborators and cannot be reimbursed in the framework of the current registry, the collection of a relatively small number of well-defined variables will be more cost-effective and will increase compliance and completeness. In addition, immediate data quality assurance procedures have to be implemented to guarantee the quality of data in a database [23]. Especially procedures concerning the data transfer from patient’s record to the data extraction sheet are especially vulnerable, and 100% source monitoring as done as routine practice in many clinical trials, is not affordable in registries. Prompt data collection close to the clinical procedures results in higher data quality [1] and makes necessary inquiries also more successful.

For the informative value of epidemiological registries, completeness is required for external (as well as internal) validity and generalizability to similar populations [1]. To achieve completeness in the ALS registry Swabia data is collected from different sources in the catchment area and also from adjuncts regions. However, the specific clinical features and severe progression of ALS makes it very possible that all patients are treated in a specialized neurological clinics or ambulances which then include the patients in this registry. Newly diagnosed ALS cases are recruited based on strict inclusion criteria and notification rules in order to collect the relevant information in the target populations with minimal work-load. All cases are reviewed by a study neurologist. Clear inclusion criteria and case definition, parsimonious data management and regular outputs with feed-back to cooperation partners as well as incentives contribute to maintain compliance and high recruitment rates. The financial reimbursement for external collaboration partners is determined according to a fair market pricing value that considers the time necessary to include the patients into the registry and the time they need to provide data usually already available as they are collected during standard routine clinical care practice. No additional procedures or measurements are in general requested and therefore the reimbursement scheme is so much lower compared to a clinical trial.

In addition, the comparison with external data resources, such as the mortality registry, provides information on the completeness of registries. The aggregated data on ALS mortality in the State of Baden-Württemberg were used for checks of completeness [24]. In the ALS registry Swabia, causes of death are identified by active follow-up. In Europe the age-adjusted incidence rate of ALS was 2.16 per 100.000 person-years [25]. The data from population registries allow identifying the number of new cases and monitoring trends in incidence over a longer period. Registries collect clinical and other information with means of observational studies, while analytical studies using registry data may, depending on the research question, open new possibilities of knowledge gain in the context of clinical registries and be conducted as cohort, case- cohort or case–control study [1]. Cohort studies are the classical method of investigating the causes of disease of frequent and common diseases (e.g. myocardial infarction or diabetes). When the prevalence is low or even rare, case–control studies are more suitable. This study type also allows the investigation of many potential risk factors including biological markers to further investigate the potential etiology and biological pathways involved. For specific research questions, more refined study designs can be applied. Nevertheless the choice of a control group is complex, because registry-based analytical studies need to control for confounders. Design strategies include matching according to specific case-characteristics for example sex, age and study region. In the ALS study Swabia, we ensured also simultaneous data collection for cases and controls. In addition, the analysis strategy has to be based on a multivariable analysis which an *a priori* strategy how to identify potential confounding factors and how to select and include them into a multivariable model [26].

For rare diseases such as ALS, registries are an approach to collect systematically data in a sufficiently large population. Sample size calculations and oversampling are methodological considerations, by which we try to achieve valid registry data. Standardization of all processes, regular quality checks and continuous training of the staff is required to ensure high quality. For the ALS registry Swabia, a standard operation handbook has been written and is used to minimize systematic errors, both in the data collection phase as well as in data management and analysis including programming errors, unclear definitions of variables or violation of the data collection protocol [23]. In addition, regular communication and publication of the data can reinforce the credibility of the registry data and increase the participation. In the ALS registry Swabia, this has been achieved by setting up an ALS-registry home-page and providing the clinicians with relevant published information [27].

The pooling of registry data will increase the power to investigate prognostic factors. In the ALS registry Swabia, we adapted the instruments to the ones applied in the EURALS consortium. Registry data can be used to recruit patients in a case–control study in order to investigate risk factors in population with heterogeneous risk [21]. The linkage to a biorepository provides additional research opportunities linking experimental with population research in order to develop diagnostic and therapeutic approaches [28,29].

Registries are an increasingly applied, powerful instrument in medical research in particular for rare diseases. The implementation of a medical registry is a long-term investigation, which requires careful considerations whether the methodological and budgetary aspects meet the purpose of the projected registry. They are a relatively cost-effective method to collect health information, which is usually already locally available, but time and effort should not be underestimated that is necessary to collect high quality data. However, in particular for the investigation of rare diseases, the implementation of registries including biological markers seems to be a promising approach to better understand the natural history of rare diseases, a better understanding of disease pathways, which then may be used as new target for treatment [21,30].

When in Ulm the decision for a registry was made, all contributors were made aware that the value and success of the registry is directly related to the quality of each component of information coming from participating health care professionals.

## Summary and conclusions

The ALS registry Swabia has been successfully implemented with excellent support of a large number of collaborating partners. In the future, the combination of a clinical registry in a defined region with a population-based control arm, allows the next generation of descriptive, epidemiological indicators, and the exploration of possible risk factors and pathophysiology (in particular of rare diseases). Registries collecting high quality information can strengthen the interdisciplinary research and present an interactive platform between patients, clinicians and scientists. However, certain methodological aspects and quality characteristics need to be taken into account to ensure the carrying capacity of the data.

## Abbreviations

ALS: Amyotrophic lateral sclerosis; EURALS: European studies on amyotrophic lateral sclerosis; HSP: Hereditary Spastic Paraplegia; MND: Motorneuron disease; PLS: Primary lateral sclerosis; PMA: Progressive Muscular Atrophy; SMA: Spinal Muscular Atrophy; SF 12: Short Form 12 Health Survey; SQoL: Subjective quality of life; HADS: Hospital Anxiety and Depression scale; FAB: Frontal Assessment Battery; MoCA: Montreal Cognitive Assessment; AMS: Achievement Motives Scale.

## Competing interests

The authors declare that they have no competing interests.

## Authors’ contributions

GN, ACL, DR were involved in the study conception and design. ACL and GN were involved in financial support. Study material or patients was provided by the members of ‘The ALS Registry Study Group’ *. Data were collected and assembled by GN, HU and AR. All authors were involved in the data analysis and interpretation. GN, HU and DR were writing the manuscript, which was finally approved by all authors. All authors read and approved the final manuscript.

## Pre-publication history

The pre-publication history for this paper can be accessed here:

http://www.biomedcentral.com/1471-2377/13/22/prepub
